# Evidence-based blood tests for monitoring adults with hypertension in primary care: rapid review, routine data analyses, and consensus study

**DOI:** 10.3399/BJGP.2025.0209

**Published:** 2026-04-08

**Authors:** Martha M C Elwenspoek, Rachel O'Donnell, Catalina Lopez Manzano, Sarah Dawson, Lewis F Buss, Katie Charlwood, Christina Stokes, Francesco Palma, Alastair D Hay, Penny F Whiting, Jessica Watson

**Affiliations:** 1 The National Institute for Health Research Applied Research Collaboration West (NIHR ARC West), University Hospitals Bristol NHS Foundation Trust, Bristol, UK; 2 Population Health Sciences, Bristol Medical School, University of Bristol, Bristol, UK; 3 Patient representative

**Keywords:** blood tests, chronic diseases, evidence-based medicine, hypertension, primary health care

## Abstract

**Background:**

Substantial variation in testing rates in adults with hypertension across UK primary care suggest that patients are not receiving optimal monitoring.

**Aim:**

To develop a minimal set of evidence-based blood tests for adults with hypertension.

**Design and setting:**

This was a rapid review, routine data analyses, and consensus study. It was set in primary care.

**Method:**

This study examined the rationale and evidence for tests recommended by guidelines or used commonly in adults with hypertension using stepwise rapid evidence reviews. A consensus group, including clinicians and patients, voted to include or exclude each test in the testing panel based on the evidence. If there was no consensus (>80%), additional evidence was sought through rapid reviews or analyses of primary care records, which was subject to further voting.

**Results:**

Sixteen routinely ordered tests were identified. The study found consistent, good evidence that estimated glomerular filtration rate (eGFR) to detect chronic kidney disease and glycosylated haemoglobin (HbA1c) to detect diabetes is beneficial for patients. The study found no or inconsistent evidence of the benefit of routinely measuring lipids, electrolytes, haemoglobin, thyroid function, clotting biomarkers, calcium, ferritin, folate acid, or vitamin B12. Good evidence was found that there is no benefit in routinely monitoring liver function, inflammation markers, or brain natriuretic peptide.

**Conclusion:**

A minimal set of evidence-based blood tests to monitor adults with hypertension was identified. This panel includes eGFR, HbA1c, potassium, and sodium. Implementing these recommendations could reduce harms associated with unwarranted variation in care. Further research is needed to clarify the role of tests with inconsistent evidence and determine the optimal frequency of testing.

**How this fits in**

Overtesting can cause patient harm, including patient anxiety, unnecessary downstream tests, referrals, and overdiagnosis, as well as wasting limited NHS resources. A minimal testing panel for patients with hypertension was developed based on the best available evidence and a consensus process. The panel includes estimated glomerular filtration rate to screen for chronic kidney disease, glycosylated haemoglobin to screen for diabetes mellitus, potassium for patients on angiotensin-converting enzyme inhibitors, and angiotensin II receptor blockers and sodium for patients on thiazide-type diuretics. Unnecessary testing could be prevented if guidelines and local protocols recommended minimal testing sets and made clear additional tests should only be added if clinically indicated.

## Introduction

Hypertension is a common long-term condition. Approximately one in four adults in the UK have hypertension.^1^ It is the leading preventable risk factor for cardiovascular disease (CVD) mortality, and morbidity.^2^ Strict management and good blood pressure control through medication or lifestyle changes reduces the risk of secondary conditions.^3^

Hypertension monitoring includes periodic measurement of blood pressure, as well as checking blood and urine. Optimal monitoring with blood tests can help monitor disease progression or treatment response, and detect secondary conditions or adverse treatment effects. However, guidance on monitoring is largely based on expert opinion,^4^ and testing rates vary substantially between general practices,^5,6^ suggesting that many patients do not receive optimal care. The majority of patients receive more tests than recommended.^6,7^

Use of laboratory tests in primary care increased fourfold between 2000 and 2015,^6^ with disease monitoring and medication monitoring accounting for around 30% and 10% of primary care testing, respectively.^8^ Overtesting is not only a waste of limited NHS resources, it can cause patient harm, including patient anxiety, unnecessary downstream tests, referrals, and overdiagnosis.^9^ Overuse of tests also increases GP workload through reviewing additional test results and costs of further investigations.

Evidence-based testing strategies that include a minimal set of tests for people with long-term conditions have the potential to ensure that people get the tests that they need and only receive additional tests when there is a clinical indication. In this article, the authors describe the development of a minimal testing panel of blood tests that are necessary for adults with hypertension using rapid reviews, routine data analysis, and a consensus process.

## Method

### Candidate tests

Candidate tests were identified based on those that are currently ordered for patients with hypertension in primary care (analysis of Clinical Practice Research Datalink [CPRD] data),^6^ that GPs say they would order for the average patient with hypertension (survey of UK primary care clinicians),^7^ and/or that are recommended by UK guidelines (review of UK guidelines).^4^

Tests were categorised by reason for testing, either to screen for secondary conditions and/or adverse treatment effects. Drug monitoring only for people on stable treatment was considered. Also, only the most commonly prescribed first-line antihypertensives were considered: angiotensin-converting enzyme (ACE) inhibitors, angiotensin II receptor blockers (ARBs), thiazide-type diuretics (TTDs) and calcium channel blockers (CCBs), and side effects listed in the British National Formulary. A list of filtering questions was determined that needed to be answered with ‘yes’ for a test to be a useful monitoring test (Box 1).

**Box 1.** Testing purpose and filtering questions

Testing purpose	Filtering question
Screen for secondary conditions	Are people with the long-term condition at an increased risk of developing the secondary condition that the test is able to detect? Is there anything the clinician can do to manage or treat this? Are there clear benefits of earlier detection or treatment?
Detect adverse treatment effects	Are people using these medications at an increased risk of developing the side effect that the test is able to detect? Is there anything the clinician can do to manage or treat this? Are there clear benefits of earlier detection or treatment?

### Rapid review methods

To help provide evidence to address these filtering questions, the following sources were looked at:

- National Institute for Health and Care Excellence (NICE) guidelines and clinical knowledge summaries (including references);
- systematic reviews on KSR Evidence (https://ksrevidence.com/); and
- primary studies on Medline, Embase, and CENTRAL.

The authors only proceeded to the next source if insufficient evidence was found.

Search strategies were developed by an information specialist (the fourth author) (Supplementary Tables S1-5). Terms were combined for hypertension, specific tests, secondary conditions, or side effects. Records were screened in two steps using Rayyan:^10^ titles and abstracts were screened excluding clearly irrelevant records, followed by full-text screening of potentially relevant records. Guidance and systematic reviews were screened by two reviewers independently. As suggested by rapid review guidelines, 20% of primary studies were double screened.^11^ Standardised data extraction forms were developed in Excel and tested by two reviewers. The inclusion criteria, number of included studies and patients, and summary estimates were extracted from reviews. The study design, population characteristics, number of included patients, and results were extracted from primary studies. Risk of bias (ROB) was assessed using ROBIS for systematic reviews,^12^ RoB 2 for RCTs,^13^ and ROBINS-E for cohort studies.^14^ One reviewer conducted data extraction and ROB assessments.

### Consensus process and additional evidence

Evidence from the rapid reviews was summarised and presented to the consensus group at an in-person meeting. The consensus group consisted of three patient representatives, four GPs (one with a joint academic appointment), one primary care nurse practitioner, one renal consultant, and two medical microbiologists. Evidence identified for each candidate test in the rapid reviews was circulated to the group ahead of the meeting (see supplementary Information S1 for the Evidence report). The quality of evidence was judged as ‘good’, ‘moderate’, or ‘weak’ (Box 2).

**Box 2.** Quality of evidence

Quality judgement	Level of evidence
Good	High-quality systematic reviews and/or high-quality studies with large sample sizes
Moderate	Systematic reviews with some quality concerns and/or several primary studies showing similar results but some may have quality concerns
Weak	A single primary study with a small sample size with or without quality concerns or several single primary studies with conflicting results

Members of the consensus group were asked to vote on whether to include or exclude each test from a minimal testing panel based on the evidence presented to them. Candidate tests were only excluded from the panel if there was no clear rationale for testing, or if the panel considered the tests failed one or more of the filtering questions based on the available information. If they could not make a decision based on the available evidence, they could also highlight the tests as requiring additional evidence. If no consensus (80%) was reached, the evidence was discussed followed by a second vote. If still no consensus was reached, the test was selected for additional evidence.

To answer specific questions that arose during the consensus meeting and to address certain gaps in the evidence, additional rapid reviews and incidence analyses using routine primary care data were conducted. These reviews addressed the following questions using the methods described above: ‘Does lipid profile testing or Q-RISK assessment increase the chance of patients following lifestyle advice?’ and ‘What is the incidence of new onset type 2 diabetes mellitus (T2DM) in a population with hypertension?’.

The incidence analyses estimated the incidence of abnormal sodium and potassium levels in people with hypertension who had normal levels at diagnosis and the methods are described here.^15^ The CPRD was used to identify people with hypertension diagnosed between 2011 and 2019 and age-, sex-, and practice-matched controls. Cox regression was used to analyse time to first abnormal blood result (sodium <135 or >145 mEq/L, potassium <3.5 or >5.5 mEq/L), to first clinically significant abnormal result (sodium <130 or >150 mEq/L, potassium <3.0 or >6.0 mEq/L).

In a second consensus meeting where the group consisted of three patient representatives, five GPs, two primary care nurses, and one renal consultant, the additional evidence was voted on using the process described above. If no consensus was reached, a third vote was held to include or exclude the test without the option of ‘additional evidence’.

### Patient and public involvement

Three patient representatives were included in the consensus group with equal voting rights to the clinicians. Two patient representatives (the seventh and eighth author) have been involved in this study from inception to completion as active members of the project management group.

## Results

### Candidate tests

Twelve testing panels (36 individual tests) were identified that are currently used or recommended to screen for secondary conditions or detecting adverse treatment effects as part of routine monitoring of people with hypertension. Five combined searches for 18 rapid reviews were conducted (see Supplementary Figures S1-5 for the PRISMA flow diagrams).

Tables 1 and 2 provide an overview of candidate testing panels, individual tests included in each testing panel, evidence available for each test, outcomes of the two consensus meetings, and the final decision on whether to include the test in the testing panel. The final testing panel included:

- estimated glomerular filtration rate (eGFR) to screen for chronic kidney disease (CKD);
- glycosylated haemoglobin (HbA1C) to screen to T2DM and to detect T2DM in those taking TTDs;
- sodium to detect hypo/hypernatraemia in patients taking TTDs;
- potassium to detect hyperkalaemia (raised potassium levels) in patients taking antihypertensive medications.

**Table 1.** Tests to screen for secondary conditions — answers to filtering questions

Blood test pane, test	Condition targeted by test	Are people with hypertension at increased risk?	Can you intervene?	Benefits of earlier detection?	CM outcomes	Final decision
**Renal function tests**	Chronic kidney disease		Yes (RR: 1 GL)	Yes (RR: 2 GL)		
eGFR		Yes (good evidence from RR: 1 GL, 3 SR, 2PS)			**CM1** 88% include	Include
Blood urea nitrogen		Yes (weak evidence from additional RR: 3 PS			CM1 more evidence needed **CM2** 90% exclude	Exclude
**HbA1C**	Type 2 diabetes mellitus	Yes (good evidence from RR: 1 GL, 1 SR, 3 PS+ additional RR post CM1: 2 SR 1 PS)	Yes	Yes	CM1 more evidence needed **CM2** 100% include	Include
**Blood electrolyte tests**						
Potassium	Hyper- or hypokalaemia	Conflicting (moderate evidence from RR of increased risk: 1 GL, 1 SR; additional IA no association)	Yes (RR: 1 GL; 1 PS)	Yes	CM1 more evidence needed **CM2** 100% exclude	Exclude
Sodium	Hyper- or hyponatraemia	No evidence from RR; Yes from additional IA	No	NA	CM1 more evidence needed **CM2** 100% exclude	Exclude
**Lipid profile**	Dyslipidaemia and assess cardiovascular risk	Yes (good evidence from RR: 1 GL, 3 SR, 2 PS)	Yes (RR: 1 GL; TT weak evidence) Additional RR on effect of lipid profile tests on behaviour (2 SR; 1PS) and TT found inconsistent evidence	Yes (1 GL)	CM1 more evidence needed **CM2** 90% exclude	Exclude
Serum cholesterol						
High-density lipoprotein						
Low-density lipoprotein						
Triglycerides						
**Liver function tests**	Metabolic dysfunction-associated steatotic liver disease (MASLD)	Unclear (weak conflicting evidence from RR: 1 SR, 2 PS)	No, other than lifestyle advice (1 GL)*	NA	CM1 100% exclude	Exclude
Albumin						
Alkaline phosphatase						
Alanine aminotransferase						
Aspartate aminotransferase						
Bilirubin						
Gamma glutamyl transpeptidase						
Total protein						
**Haemoglobin (full blood count)**	Anaemia	No evidence	Yes (RR: 1 GL)	Yes (RR: 1 GL)	CM1 88% exclude	
**Haematinics**	Anaemia and deficiency type	No evidence	Yes (RR: 1 GL)	Yes (RR: weak evidence 1 PS)	CM1 88% exclude	Exclude
Vitamin B12						
Serum ferritin						
Folate tests						
**Clotting tests**		No evidence	No evidence	No evidence	CM1 88% exclude	Exclude
Prothrombin time	Bleeding disorders					
Partial thromboplastin time	Hyper- or hypocalcaemia					
Thrombin time	Deficiencies and anaemias					
**Bone profile**	Serum calcium and phosphorus levels, vitamin D deficiency, parathyroid problems		Yes (RR: 1 GL)	No evidence	CM1 88% exclude	Exclude
Serum inorganic phosphate		No evidence				
Calcium/adjusted calcium		No evidence				
Vitamin D		Yes (weak conflicting evidence from RR: 1 PS)				
Alkaline phosphatase		No evidence				
Parathyroid hormone		No evidence				
**Thyroid function tests**	Hyper- or hypothyroidism	No evidence	Yes (RR: 1 GL)	No evidence	CM1 86% exclude	
Thyroxine						
Free thyroxine						
Thyroid stimulating hormone						
Triiodothyronine						
**B-type natriuretic peptide**	Heart failure	Yes (weak evidence from RR: 1 GL, 1 PS).	Yes (RR: 1 GL)	No evidence	CM1 88% exclude	Exclude
**Inflammatory markers**	General marker for inflammation	Yes (good evidence)	No	NA	Excluded before CM1 owing to lack of consensus on treatment	Exclude
C-reactive proteinand erythrocyte sedimentation rate						

CM = consensus meeting. CM1 = first CM. CM2 = second CM. eGFR = estimated glomerular filtration rate. GL = . HbA1C = glycosylated haemoglobin. IA = . PS = . RR = . SR = .

**Table 2.** Tests to screen for adverse treatment effects of antihypertensives for patients on stable treatment — overview of answers to filtering question and consensus meeting outcomes

Test panel, test	Condition targeted by test	Are people on these medications at increased risk?	Can you intervene?	Benefits of earlier detection?	CM outcomes	Final decision
**HbA1C**	Type 2 diabetes mellitus					
		No (good evidence) for ACE, ARBs, CC			CM1: 89% exclude	Exclude
		Yes for TTDs (good evidence)	Yes (change medications)	Yes (change medications sooner)	CM1: 89% include for TTDs	Include
**Renal function tests**	Kidney injury	No evidence for CCBs or TTDs ACEis and ARBs: inconsistent (moderate evidence from RR: 2 SR)	Yes (change medications)	Yes (change medications sooner)	CM1 more evidence needed **CM2** 80% exclude	Exclude
eGFR						
Urea						
**Blood electrolyte tests**						
Potassium	Hyper- or hypokalaemia	ACEis and ARBs: Yes (good evidence) No evidence for CCBs, RRD, or TTDs	Yes (change medications)	Yes (change medications sooner)	CM1 more evidence needed **CM2** 80% include	Include
Sodium	Hyper- or hyponatraemia	Yes for TTDs	Yes (change medications)	Yes (change medications sooner)	CM1: not considered **CM2**: 80% include	Include
**Liver function tests**	Liver disease and damage	No evidence; consensus that people with HTN not at increased risk of these side effects than others taking drugs			CM1 100% exclude **CM2** 100% exclude	Exclude
Albumin						
Alkaline phosphatase						
Alanine aminotransferase						
Aspartate aminotransferase						
Bilirubin						
Gamma glutamyl transpeptidase						
Total protein						
**Haematinics**	Anaemia and deficiency type	No evidence			CM1 100% exclude **CM2** 100% exclude	Exclude

ACEi = angiotensin-converting enzyme inhibitor. ARB = angiotensin II receptor blocker. CM = consensus meeting. CM1 = first CM. CM2 = second CM. CCB = calcium channel blocker. eGFR = estimated glomerular filtration rate. HbA1C = glycosylated haemoglobin. HTN = . RR = . RRD = . SR = . TTD = thiazide-type diuretic.

### Tests with good evidence and rationale

There is consistent, good evidence that patients with hypertension are at higher risk of CKD than the general population.^16–20^ The consensus panel agreed that screening for CKD using eGFR is beneficial.

There is consistent, good evidence that patients with hypertension are more likely to have T2DM, they are more likely to develop T2DM after diagnosis of hypertension, and increases and decreases in blood pressure are related to increased and decreased risk, respectively, of developing T2DM.^19–22^ Early detection and treatment of T2DM is beneficial,^23–27^ so the consensus panel agreed that regular monitoring of HbA1c is justified.

Finally, good evidence was found that ACE inhibitors and ARBs increase the risk of hyperkalaemia,^28,29^ and that TTDs increase the risk of T2DM,^30,31^ and there was consensus to include potassium and HbA1c for people on these medications, respectively.

### Tests with unclear evidence or rationale

Inconsistent evidence was found whether hypertension increases the risk of hyper- or hypokalaemia. A systematic review found a higher prevalence of hyperkalaemia in people with hypertension compared with people who do not have hypertension.^32^ Analyses of routine data showed that patients with hypertension with normal potassium levels at diagnosis have a similar incidence of abnormal levels compared with controls.^15^

No evidence was found on the prevalence of abnormal serum sodium levels in patients with hypertension. The study’s additional analyses of routine data showed that patients with hypertension who had normal sodium levels at diagnosis were at higher risk of developing abnormal levels compared with controls.^15^ However, this may have been caused by medications rather than hypertension itself, which was not taken into account in this analysis. Thus, this did not provide strong evidence that patients with hypertension who do not take medication benefit from sodium monitoring.

The consensus panel concluded that sodium and potassium did not need to be monitored to screen for secondary conditions (Table 1); however, they were included in the final panel for medication monitoring (Table 2). As most laboratories automatically report sodium and potassium when eGFR is requested, these recommendations are unlikely to alter clinical practice, even for patients not taking regular medications.

Lipid profile is used to calculate the Q-RISK score to assess cardiovascular risk. There is good, consistent evidence from multiple systematic reviews that hypertension increases the risk of CVD,^33–35^ and a large population-based survey has shown that hypertension is associated with higher lipid levels. However, the Q-RISK score can be calculated without lipids or with a single measurement of lipids at diagnosis. GPs can manage this risk with lifestyle advice, tight blood pressure control, and statins. The consensus group suggested that regular lipid monitoring may motivate people to adhere to lifestyle advice, but evidence that the test result itself could prompt a change in lifestyle is inconsistent and weak, suggesting that this is not a good rationale for monitoring. Frequent lipid monitoring is often misleading because of biological and analytical variation. The minimum change needed between two lipid tests to be statistically meaningful (the reference change value) is high; 11–20% for total cholesterol and 21–30% for high-density lipoprotein and low-density lipoprotein. True changes in cholesterol typically take 3–5 years to exceed this 'background noise'. Given the available evidence, the consensus group was not convinced that regular monitoring after initial assessment of lipid profile at diagnosis is beneficial and excluded the test.

No evidence was identified on whether hypertension increases the risk of conditions detected by haemoglobin, thyroid function, clotting, calcium, ferritin, folate acid, and vitamin B12 tests. The consensus group decided that in the absence of evidence and a known biological mechanism, these tests should be excluded given the potential for harm from unwarranted testing.

No evidence was identified linking antihypertensive drugs to abnormal liver function or anaemia once patients are on stable treatment. No evidence was identified on eGFR or potassium levels in people taking CCBs or TTDs. In the absence of evidence of clinical need, the consensus group decided to exclude these tests for the purpose of monitoring adverse effects of these medications.

### Tests with evidence against routine use

Liver function tests can help to detect metabolic dysfunction-associated steatotic liver disease (MASLD), but it is unclear if people with hypertension are at greater risk because the evidence is weak and contradictory.^36–38^ Importantly, the management, namely lifestyle advice, would be similar to lifestyle advice for hypertension management. There was consensus that monitoring liver function does not benefit patient outcomes because of a lack of treatments for MASLD, although this may change in the future.

C-reactive protein (CRP) and erythrocyte sedimentation rate (ESR) were excluded because there is no guidance or agreement on how to treat low-grade inflammation. Brain natriuretic peptide was excluded because it was not developed as a monitoring test and was considered to be too inaccurate.

Good evidence was found that ACE inhibitors and ARBs are renoprotective and reduce the risk of developing T2DM,^30,31^ so regular additional monitoring of eGFR and HbA1c in people on these medications is not supported by the evidence, although eGFR should be checked before and after ACE inhibitor initiation and dose increments to detect renal artery stenosis. Good evidence was also found that people on CCBs are not more likely to get T2DM than people receiving placebo,^30,31^ so additional monitoring of HbA1c in people on CCBs is not warranted either.

## Discussion

### Summary

A minimal evidence-based testing panel of blood tests for adults with hypertension was identified. This panel includes eGFR to screen for CKD, HbA1c to screen for T2DM, potassium for patients on ACE inhibitors and ARBs, and sodium for patients on TTDs.

Also identified was a list of tests where evidence is absent or inconsistent: lipids, electrolytes, haemoglobin, thyroid function tests, clotting tests, calcium, ferritin, folate acid, or vitamin B12 to screen for secondary conditions; liver function and anaemia tests for adverse effects caused by antihypertensives, and eGFR or potassium levels for adverse effects caused by CCBs or TTDs.

Finally, good evidence was found that there is no benefit in routinely monitoring liver function, inflammation markers such as CRP and ESR, or brain natriuretic peptide and that monitoring eGFR and HbA1c to detect side effects of ACE inhibitors, ARBs, and CCBs is not warranted. The consensus group agreed that in the absence of evidence or clinical need these tests should not be offered to people with hypertension.

### Strengths and limitations

The main strength of this study is that established and systematic methods were used to identify evidence. To address some of the gaps in the literature, routine data analyses were conducted using a large cohort of primary care patients who are largely representative of the UK population regarding age, ethnicity, and sex. Another strength is the inclusion of a diverse consensus group to judge the evidence and make the final decisions on including or excluding tests for the minimal testing panel.

This study also has several limitations. For pragmatic reasons, rapid review methods were used and, as a result, relevant evidence may have been missed or errors made in data extraction and the ROB assessment as these were not checked by a second reviewer. In this study the authors did not consider the accuracy, biological variation, or optimal thresholds of each tests when considering the evidence, which may be important to consider. Also, differences in study samples were not assessed and the focus was on the 'typical' patient with hypertension; future research could explore the applicability of the current findings to diverse at-risk groups. The methods used in the current study are dependent on the availability of existing evidence, which continues to evolve; future research, including randomised controlled trials, would be valuable in addressing current gaps and strengthening the evidence base. However, the few tests that were included in the final panel are well established. A health economic evaluation was not included in this analysis and the optimal frequency of monitoring was not looked at, which are important next steps to address.

### Comparison with existing literature

Similar to current UK guidelines, who recommend regular renal function testing, the authors of the current study recommend regular testing of eGFR. Also recommended is the monitoring of electrolytes (sodium and potassium) for people on specific medications. Many laboratories will include electrolytes automatically when ordering eGFR, and so these are likely to be ordered for everyone with hypertension, not sure how those on these medications. The authors of the current study recommend regular monitoring of HbA1c to detect new-onset T2DM in people with hypertension. This is not mentioned in the NICE Hypertension guidelines,^39^ although it is suggested in the public health diabetes prevention guidelines.^40^

In practice, the majority of patients receive more tests than are in the current study’s minimal testing set or that are recommended by guidelines. Many patients with hypertension receive annual monitoring of full blood count, lipid profile, and liver function.^6^ A survey among GPs suggested that this is not just because of clinical need or comorbidities, as it showed that 19–52% of GPs would routinely order lipids, liver function tests, and full blood count for an average patient with hypertension.^7^

### Implications for research and practice

The evidence-base for the majority of tests that are routinely ordered for adults with hypertension is weak or absent. In contrast, there is good evidence of the harms of overtesting, so in the absence of evidence, the authors of the current study suggest tests should not be offered routinely. National guidelines should reflect this and local protocols need to be updated to avoid unnecessary testing. New guidance and local protocols need to make clear that additional tests can be added if clinically indicated, but should not be included for routine monitoring purposes. Educating GPs and staff requesting tests about the harms of overtesting may help avoid tests being added ‘just in case’ or ‘because we have always done this’.

The current study’s research suggests that the minimal testing panel is beneficial for most patients with hypertension. However, there may be exceptions. In some patients, the benefit of monitoring may not outweigh the harms of testing. For instance, in older patients who are frail and have multiple conditions and limited life expectancy, regular monitoring may do more harm than good. The final decision to monitor, what to monitor, and how often should still be made on an individual basis and in collaboration with the patient.

Many tests are offered annually for practical reasons but it is unclear if this is the best frequency. Future research should consider the optimal frequency of monitoring. As randomised trials on monitoring are challenging, because of the long follow-up time that is needed to show the benefit of monitoring, this may be partially addressed using health economic modelling.

## Data sharing

The data from rapid reviews and the consensus meetings that support the findings of this study are available within this paper and supplementary files. A small part of this study is based on data from the Clinical Practice Research Datalink (CPRD) obtained under licence from the MHRA and NIHR. The data are provided by patients and collected by the NHS as part of their care and support. CPRD data can be accessed via the MHRA: https://www.cprd.com/.
